# Anxiety Disorders, Anxious Symptomology and Related Behaviors Associated With Migraine: A Narrative Review of Prevalence and Impact

**DOI:** 10.1007/s11916-024-01312-9

**Published:** 2025-01-29

**Authors:** Jaroslava Raudenská, Tomáš Macko, Šárka Vodičková, Dawn C. Buse, Alena Javůrková

**Affiliations:** 1 Department of Nursing, 2Nd Faculty of Medicine, Charles University, Prague, Czech Republic; 2 Department of Clinical Psychology, University Hospital Kralovske Vinohrady and 3rd Faculty of Medicine, Prague, Czech Republic; 3 Department of Neurology, Albert Einstein College of Medicine, Yeshiva University, Bronx, NY USA

**Keywords:** Migraine, Anxiety, Fear, Avoidance, Anxiety sensitivity, Phobias, Cephalalgiaphobia, Cogniphobia, Generalized anxiety disorder

## Abstract

**Purpose of Review:**

The purpose of this study was to review the literature on the relationship between migraine, anxiety and related disorders, anxious symptomology and related behaviors.

**Recent Findings:**

Generalized anxiety, other anxious disorders and migraine are comorbid. In addition, anxious symptomology and behaviors are common in people with migraine even if they do not meet diagnostic criteria or threshold. Anxiety including diagnosed disorders such as generalized anxiety, phobias, panic disorder, as well as behaviors such as catastrophizing, avoidance behaviors, and higher fear of headache/migraine or anxiety sensitivity are comorbid and/or common in migraine. Anxiety is associated with negative outcomes such as migraine progression, medication overuse, stigma and migraine-related disability.

**Summary:**

The association between migraine, anxiety, and fear and avoidance behaviors has an extensive empirical basis. Awareness of the high prevalence of comorbidity and symptomology as well as the negative outcomes associated with anxiety and related symptoms and behaviors is important in the comprehensive management of people with migraine. Better understanding the relationship between migraine and anxiety symptoms and behaviors and their effects on outcomes is essential to provide more effective treatment for people with migraine. The review emphasizes the necessity of screening and more comprehensive evaluation in patients with migraine using psychological diagnostic tools. Thus, prevention and management of anxiety, fear, and anxiety-related behaviors in the context of migraine management may be considered an essential treatment goal and strategies may include non-pharmacological and pharmacological approaches.

## Introduction

Globally, migraine is the leading cause disability among all neurological conditions [1] and one of the 20 most common causes of disability in the world [2]. It is well established that anxiety is comorbid with migraine [3–7] and is associated with several undesirable outcomes [7–12]. In additional to generalized anxiety disorder, related anxiety disorders such as specific phobias, social phobias, panic attacks and other disorders are also comorbid with migraine. Anxious symptoms and resulting behaviors such as catastrophizing and avoidance behaviors also occur at high rates among people with migraine, and may manifest as fear, avoidance, and ictal and interictal burden. Fear in people with migraine can be manifested in the form of anticipatory fear of medical and social factors, fear of the next migraine attack (pre-emptive or interictal anxiety), fear of causing or exacerbating headache or pain (cephalalgiaphobia, cogniphobia) or anxiety sensitivity [13–15]. Anxiety and diseases associated with anxiety contribute to the higher disability and treatment costs in people with migraine [16, 17]. Disorders connected to anxiety are associated with increased risk of chronic headache and chronic migraine [7, 18, 19]. The connection between anxiety, fear and migraine may suggest that psychological treatment can reduce or modify some of the negative outcomes in migraine. That is supported by functional magnetic resonance imaging (fMRI), which shows that cognition, emotions and experiences change the ways the brain processes pain inputs [20].

## Methods

The search for scientific studies for this review was conducted from March until July 2022 using the Ebscohost, PubMed and Cochrane databases as well as Goggle scholar and the Sage Journals website.

The following keywords and terms were used: migraine & anxiety, migraine & fear, migraine & anxiety disorders, fear of pain & migraine, anxiety sensitivity & migraine, cephalalgiaphobia & migraine, cogniphobia & migraine. A secondary search supplemented the primary search through the bibliography of already acquired studies. All chosen articles were reviewed by title, abstract, and the article itself if there was no clear title or abstract. Inclusion criteria were as follows:

1. Article types including original data articles, meta-analyses or systematic reviews.
2. The article referred to migraine, or pain associated with migraine and the relationship between migraine and concepts related to anxiety (e.g., comorbidity of anxiety disorders, fear, personality traits, phobias etc.).
3. The article was in English.
4. The full article was available.

## Results

**Fig. 1:**
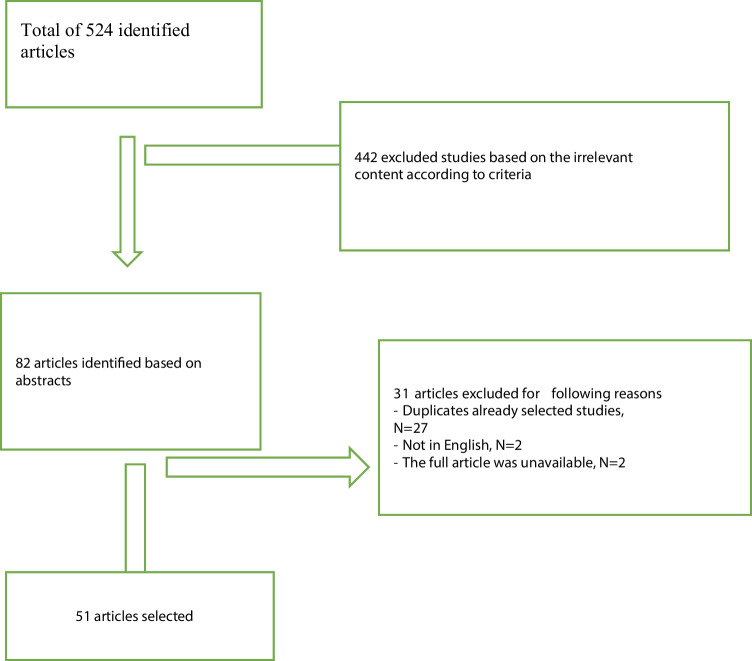
Flow diagram: Process of selected studies

Overall, 51 studies that met all criteria were identified from 524 results (Fig. 1.). Articles that met the search criteria were published between 1986 and 2022. Half of the publications (25 studies) were published between 1986–2012 [5, 7, 18, 21, 22, 26–28, 30, 31, 38, 39, 49, 50, 54, 55, 59, 60, 63, 64, 66–68, 73, 77] and half (26 studies) between 2013–2022 [4, 29, 32, 35–37, 40, 44, 46–48, 51, 53, 61, 62, 65, 69, 72, 74, 75, 78, 79, 80, 103–105]. The relationship between anxiety or fear was investigated from different perspectives. The most commonly reported topics were related to personality characteristics (e.g. anxiety traits and personality types) and psychiatric diagnoses and symptoms (phobias, panic disorder etc.). There were four epidemiologic studies [27, 28, 65, 67] identified in these areas as well as one meta-analysis [37], and two systematic reviews [4, 78]. However, the relationship between migraine and sensitivity to anxiety and the relatively new concepts of cogniphobia, kinesiophobia and cephalalgiaphobia have not been studied sufficiently [68, 69, 72–75, 77, 80]. The overview of studies is summarised in Table 1. We have divided the studies into the following topic areas: Comorbidity and prevalence of migraine and anxiety disorders [4, 7, 18, 21, 22, 26–32, 35, 65–67, 78, 79], Fear of pain and avoidance behavior in migraine [5, 36–40, 44], Migraine, personality traits/disorders associated with anxiety and fear [45, 53–55, 60–64], Migraine and Anxiety sensitivity [46–51], Migraine and Phobias: Cephalalgiaphobia, [68, 69, 80] Cogniphobia and Kinesiophobia [72–75, 77]. Included studies are presented in Table 1.

**Table 1:** The overview of studies

Migraine and Anxiety disorders	2 systematic reviews 4 epidemiologic studies 8 empirical studies 6 reviews 2 scientific articles	Dresler et al. [4] Guidetti et al. [18] Radat & Swendsen [21] Breslau [22] Smitherman et al. [7] Breslau et al. [26] Breslau et al. [27] Breslau & Davis [28] Zhang et al. [29] Green [30] Baldacci et al. [31] Noseda et al. [32] Chen et al. [35] Merikangas et al. [65] Peroutka et al. [66] Swartz et al. [67] Karimi et al. [78••] Kumar et al. [79] Buse et al. [103] Buse et al. [104] Minen et al. [105]
Fear of pain and avoidance behavior in migraine	1 meta-analysis 5 empirical studies 1 scientific article (a chapter in a monograph)	Philips & Jahanshahi [5] Helsen et al. [36] Zale et al. [37] Asmundson et al. [38] Hursey & Jacks [39] Ruscheweyh et al. [40] Black et al. [44]
Migraine, Personality traits and disorders associated with anxiety and fear	1 meta-analysis 5 empirical studies 3 reviews	Mongini et al. [45] Davis et al. [53] Balottin et al. [54] Lake et al. [55] Sances et al. [60] Bottiroli et al. [61] Galli et al. [62] Frith [63] Tani et al. [64]
Migraine and Anxiety sensitivity	5 empirical studies 1 scientific article	Harvard Health Publishing [46] Farris et al. [47] Farris et al. [48] Asmundson & Taylor [49] Norton & Asmundson [50] Smitherman et al. [51]
Migraine and Phobias: Cephalalgiaphobia	2 empirical studies 1 validation study	Peres et al. [68] Giannini et al. [69] Klan et al. [80]
Cogniphobia and Kinesiophobia	2 empirical studies 1 pilot study 2 scientific articles/presentations	Seng [72] Todd et al. [73] Seng & Klepper [74] Martin et al. [75] Suhr & Spickard [77]

### Migraine and Anxiety Disorders: Comorbidity Prevalence and Impact

Pain has a protective character, it protects the individual from danger, it is normal for people to be afraid or afraid of pain. A phobia is an irrational and disproportionate fear of an object, activity, or situation that is not normally dangerous. Psychiatric disorders are comorbid with migraine including anxiety disorders [21, 78, 79]. According to observational studies, anxiety disorders occur in 20–75% of people with migraine [22]. Rates are higher in clinic populations relative to population samples and rates increase with higher headache day frequency and higher average headache pain intensity, suggesting a connection between anxiety disorders and chronic states [7, 21–25, 78, 103–105]. People with migraine are 3–6 times more likely to have panic disorder than people without migraine, with some studies suggesting that they are nine times more likely [7, 23]. There is also have a 5.5-fold increased risk for generalized anxiety disorder, a fivefold increased risk of obsessive–compulsive disorder (OCD), and 2.5 times higher probability of specific phobia(s) people with migraine [7, 27, 28].

Relationships between anxiety disorders and migraine are bidirectional; the incidence of migraine increases the risk of anxiety and vice versa [7, 18, 26] or there may be a shared underlying mechanism. The comorbidity of anxiety disorders and migraine may be explained by shared genetic and environmental impacts, neurotransmitter systems (i.e., serotonergic dysfunction), fluctuations in ovarian hormones or disturbed regulation of the hypothalamic–pituitary–adrenal **(**HPA) axis among other hypotheses [4, 7, 10, 11].

Anxiety disorders and migraine are perceived as autonomous regulations that could exacerbate pain intensity and disability [4, 30]. Furthermore, anxious symptoms (similar to depressive symptoms) may be associated with higher sensitivity to the evocation of migraine, and/or may lower the attack threshold. Altered pain sensitivity due to anxiety (or depressive symptoms) can be a result of a lowered pain threshold and increased cortical arousal caused by neurolimbic dysfunction [4, 31].

The relationship between chronic migraine and serotonin and dopamine abnormalities has been observed in animal models. Treatment associated with these changes leads to improvements in anxiety and chronic migraine [4, 29]. Anxiety can modulate migraine symptoms by affecting trigeminovascular thalamic neurons that transmit information related to migraine and headaches [4, 31]. The negative correlation between the size of hippocampal areas and the degree of anxiety is interesting to consider in the relationship between migraine, pain, and anxiety [4, 32]. It is also necessary to consider in development of panic disorder interoceptive conditioning and concepts of fear of pain, sensitivity to anxiety or avoidant behavior that can be considered as vulnerability factors of migraine [4].

### Fear of Pain and Avoidance Behaviors in Migraine

Fear of pain is a concept that describes different forms of fear associated with pain [33, 34]. We can differentiate fear of pain according by the anticipated source of the threat, e.g., fear of the occurrence and prolongation of pain, physical activity, and repeated evocation [35]. Phobias in relation to pain are defined as chronic, irrational, fear of somatic pain or extreme discomfort. Phobia of pain, or algophobia, can cause worry, panic or depression when thinking of pain [35–37].

A meta-analysis based on 46 studies (*N* = 9579) with patients with different forms of chronic pain found that fear of pain is positively related to degree of disability with a medium to large effect size among all demographic groups and types of pain. These findings are consistent with the cognitive-behavioral and fear avoidance models, which suppose that fear leads to a greater avoidance and from a long-term perspective to a more significant disability [37]. Research has not been conducted testing this in migraine. However, some studies suggest that fear of pain increases in patients with more frequent or continuous “persistent” headaches [38, 39, 100–102]. Therefore, the role of fear of pain should also be considered in terms of progression to more frequent migraine or maintaining high frequency episodic or chronic migraine, although when someone experiences constant or continuous pain there is no interictal period in which to fear the onset of pain [5, 38].Through the negative reinforcement, aka operant conditioning, over time patients experience fear activation merely from the expectation of pain. Acute reductions in anxiety and fear then act to maintain this cycle, perpetuating anxiety sensitivity through negative reinforcement.

The fear-avoidance model (FA model) describes the relationship between fear and aggravation of pain [40, 41]. It explains how increased fear associated with pain expectations leads to avoidant behaviour, deterioration, worsening or intensifying of the condition, depression, and greater disability [42]. It is classified as a psychological mechanism contributing to the transition from acute to chronic pain. In contrast, the avoidance-endurance model (AE model) offers explanations that assume that chronicity in pain leads to continuous overloading, resulting in aggravation of pain [43]. These two models were originally developed in musculoskeletal pain; however, they might also apply to migraine since both forms of behavior are frequently observed in people with migraine and other severe headaches [40].

Fear of migraine can discriminate between people with pain (higher fear of pain) and people without pain and between patients with tension pain and migraine (higher fear of pain) [44]. Concurrently, fear of pain predicted severity of headache, frequency, and explained more variability in disability than a combination of gender, anxiety and depression. Although disability strongly correlated with the severity of headache, fear of pain partially mediated this correlation [44].

A comparative study conducted on 98 women which employed Cloninger's temperament and character inventory (TCI) revealed significantly higher scores in harm avoidance and persistence that were associated with the presence of migraine [45].

### Migraine and Anxiety Sensitivity

Anxiety sensitivity refers to the extent of beliefs that anxiety symptoms or arousal can have harmful consequences. In this case, individuals misappraise anxiety and bodily sensations in terms of their physical, social, or cognitive consequences, catastrophizing the potential outcomes [46]. It is hypothesized that anxiety sensitivity may contribute to the comorbidity of migraine, anxiety and depressive symptomology and also to greater migraine severity and it was described in 100 study participants [47].

Results of a different study of 100 women with migraine suggest that anxiety sensitivity and cognitive concerns were significantly associated with a higher probability of avoiding moderate and intense physical activity [48]. The same analysis found that anxiety sensitivity was associated with more intense pain during migraine. Empirical evidence partially supports Amundson and Taylor's model [49] described in patients with chronic muscle pain. The model supposed that avoidant behavior which is a part of the maladaptive process that maintains and promotes pain, positively correlates with fear of pain; hence, fear of pain indirectly contributes to pain chronification. Additionally, anxiety sensitivity may indirectly contribute to chronification since it exacerbates fear of pain [50]. Of note, chronic pain refers to pain which occurs over a long period of time (for example six months or longer) while chronic migraine refers to having a diagnosis of migraine and experiencing headache on 15 or more days per month, of which eight or more are linked to migraine, so chronic migraine in essence refers to increased frequency while chronic pain refers to longer duration.

A case study's findings demonstrated a positive correlation between the severity of pain and fear of pain, similar to a correlation between fear of pain and anxiety sensitivity. As expected, a strong relationship between fear and avoidant behavior was identified via structural equation modeling [50] the prediction of a direct significant loading of anxiety sensitivity on fear of pain and headache severity had a direct loading on fear of pain. The presumed relationship between the severity of pain and avoidant behavior was not entirely consistent, authors explain this relationship with the specifics of muscular pain and headache [50].

A cross-sectional study conducted on a sample of 2,350 participants analysed the relationship between anxiety sensitivity and assumed negative consequences. Anxiety sensitivity was a reliable variable for distinguishing between people with and without headache. Participants with chronic migraine and episodic migraine with aura achieved the highest scores on the anxiety sensitivity scale. Moreover, it was responsible for the unique dispersion of symptoms of depression and anxiety [51].

Recently, a study conducted with 100 women with migraine revealed similar results indicating a significant relationship among anxiety sensitivity and severity of anxiety, depressive symptoms and fear of pain. The cognitive aspect of anxiety sensitivity significantly correlated with the length of migraine attacks, intensity of pain, pain avoidance, and more frequent use of non-prescribed drugs [47].

These studies all identified relationships in cross sectional analyses. Potential causality, directionality, and the ability to modify outcomes needs to be explored further.

### Migraine, Personality Traits (Neuroticisim), Personality Disorders Associated with Harm Avoidance, Anxiety/Depressive/Adjustment

Research into identifying specific personality traits associated with migraine may be stigmatizing. Cross sectional results should be interpreted with caution and great care should be taken to not imply blame to the person with migraine for causing or maintaining migraine due to certain trait or state-based personality variables. Nonetheless, there is a body of literature which has examined specific personality traits, primarily in a cross-sectional manner to identify associations. Neuroticism as a trait has been one focus of this research. Neuroticism is also characterized as a trait with tendency of high emotional instability or negative emotionality. Neuroticism is define as an tendency toward abroad range of negative feelings including distress, anxiety, self-doubt, and other negative feelings (especially impulsiveness, vulnerability, suggestibility, affective lability, separation anxiety, hostility, suspiciousness, and perseveration). In the mid-twentieth century, psychologists identified five broad personality traits including extraversion, agreeableness, openness, conscientiousness, and neuroticism. All personality traits are posited to exist on a spectrum [52]. The results of a systematic review suggest that migraine is associated with neuroticism [53]. However, the relationship's direction is unclear, as is the extent to which neuroticism reflects stable trait characteristics or is only a passing state or symptom of somatic problems [53]. The specific mechanism of action triggering a patient’s anxiety could be a trait or state. A 2012 meta-analysis concluded that, compared to children without migraine or tension type headache, children with migraine and/or tension type headache displayed more anxiety disorders and inhibited behavior and also aggressive and antisocial signs [54]. Treatment of migraine can also be complicated by other personality disorders [55]. Borderline personality disorder has been associated with adverse impacts and outcomes for people with migraine, including worse response to treatment and the risk of medication overuse [53, 56, 57]. Strategies for successful clinical management include shared goal setting and agreement with explicit contracts for treatment [58].

Another comparative study comparing people with migraine using the MMPI-2 questionnaire (Minnesota Multiphasic Personality Inventory®−2) found that patients with episodic headache and medication overuse scored significantly higher in hypochondria, depression and hysteria (and lower in ego power and dominance). Furthermore, patients with episodic headache and medication overuse had higher scores in hypochondria and health problems than patients with episodic headache without medication overuse [59, 60].

The study from 2018 compared the group of patients with chronic migraine with patients with episodic migraine (EM) found that patients with less chronicity experienced less traumatic and stressful situations (physical and emotional) inchildhood and the present [61]. A clinic-based study of 80 chronic migraine (CM) patients, 44 EM patients and 67 controls found statistically significant differences between all three groups on their ability to express their feelings in words, with the group with CM having the highest score on alexithymic features [62]. Alexithymia refers to a condition when an individual has difficulty in expressing their feelings or emotions in words and difficulty in distinguishing between feelings and the physical sensation of emotional arousal. Some evidence indicates that alexithymia is associated with fear in general [63, 64, 97]. The previously described research results suggest that particularly chronic migraine and medication overuse are connected with selected features associated with anxiety. These results are aligned with a Zurich study that found that anxiety disorders often precede migraine [65]. Epidemiologic cohort study of 27- and 28-year-olds people in Zurich found the prevalence of migraine of 13.3% in population. The association between migraine and the anxiety disorders was strong comparing other affective disorders.

### Migraine and Phobias

Phobias can occur in up to half of people with migraine [65]. They are genetically associated through the DRD2 dopamine receptor allele [66]. A Baltimore area study, conducted in the 1980s with a follow-up after 12 to 15 years, included history focusing on headaches and follow-up headache ratings. The association between migraine and psychiatric diagnoses was estimated. In the at-risk population of 1,343 people, there were 118 cases of migraine. A Baltimore study concluded that the presence of a phobia at baseline predicted incidence of migraine (odds ratio, 1.70; 95% confidence interval, 1.11–2.58) [67]. As already proposed in the section on anxiety sensitivity, there is a certain overlap in avoidance behavior in both phobias and migraine, which has led to the use of the term cephalalgiaphobia [68].

### Cephalalgiaphobia

Cephalalgiaphobia is a the fear of a headache or migraine attack which may lead patients to take analgesics in the absence of pain to prevent headaches, possibly leading to medication overuse, as well as being associated with anxiety, avoidance behaviors, fear or making plans, and negative impact on quality of life [69, 80]. The prevalence of cephalalgiaphobia in the chronic migraine population may be up to 60% [68]. Giannini's et al. pilot prospective cohort study [69] of on 126 people with chronic migraine (CM) with medication overuse showed that they had significantly higher scores of cephalalgiaphobia than CM patients without medication overuse. A higher score of cephalalgiaphobia was also associated with a higher frequency of migraine. One explanation is that anticipatory anxiety leads patients to use analgesics earlier. This lowered threshold for analgesic use may lead to a vicious circle of headache and acute medication overuse [68]. However, data from enough patients and a meta-analytical or systematic summary are currently unavailable on this topic.

### Cogniphobia and Kinesiophobia

Cogniphobia refers to fear and avoidance of mental exertion due to a fear of a headache or migriane attack while kinesophobia refers to a parallel fear and avoidance of physical movement and activity due to fear of pain injury or re-injury [70–72]. It is also defined as dysfunctional coping style.Experts and patients alike look for triggers that may evoke migraine attacks. Some people with migraine believe or worry that intense thinking is an attack trigger for them and develop cogniphobia [72]. Cogniphobia, was first described in relation to posttraumatic headaches [73] but can also be related to the fear or inciting or exaccerbating a migraine attack [74]. Fear and the associated avoidance of triggers may be unproductive in the treatment of migraine, and conversely, may contribute to disability and reduce the range of typical daily activities [72, 75, 76]. A study of 74 young adults with migraine and headaches showed an association between cogniphobia and more catastrophizing, anxiety, and pain avoidance [77]. Similarly, a pilot study for the development of a tool for measuring cogniphobia in headaches Cogniphobia Scale for Headache Disorders (CS-HD) conducted on 80 patients with migraine suggested that a higher cogniphobia score is related to clinically significant symptoms of anxiety (*ρ* = 0.37, *p* = 0.001) [74].

### Patient Reported Outcome Measures of Anxiety in General and Avoidance Among People with Migraine/Headache

There are several patients reported outcome measures (PROMs) to measure common forms of avoidance in people with migraine or other headache disorders. The Headache Trigger Sensitivity and Avoidance Questionnaire (HTSAQ) assesses the extent to which people with migraine and other headache conditions believe they are sensitive to a range of triggers. It also assesses the extent to which they avoid these perceived triggers. It occurs in both full and abbreviated form [81, 82]. The Cogniphobia Scale for Headache Disorders (CS-HD) assesses fear and avoidance of mental effort perceived as causing headache [74]. The Headache Acceptance Questionnaire (HAQ) assesses acceptance of headache and avoidance of activities in an attempt to avoid headache or migraine attacks [83]. The items measure the degree to which respondents avoid activities. Low avoidance is termed acceptance. The HAQ can be used to identify psychological responses to headache symptoms, finding treatment targets, particularly reducing avoidance.

There are also a range of PROMs developed for generalized anxiety disorder (GAD) and other forms of anxiety including the GAD-7, GAD-2 [84], as well as the Patient Health Questionnaire- 4 item (PHQ-4) [85, 86] which is a screener for anxiety and depression that includes both the GAD-2 and the PHQ-2, the Hamilton anxiety rating scale (HAM-A) [87], the State Trait Anxiety Scale (STAI) [88], and PROMs that measure related symptoms such as the Pain Catastrophizing Scale [89, 90].

## Discussion

Anxiety and a range of anxious disorders are comorbid with migraine and associated with several undesirable outcomes. In addition, anxious symptomology and behaviors are common including fear and avoidance behaviors. Anxiety prevalence is higher in people with migraine compared with the general population and rates increase as monthly headache day frequency increases. Anxious and fear-related problems such as cephalalgiaphobia, migraine sensitivity and cogniphobia are also associated with anxiety in people with migraine. There appears to be a bidirectional relationship between anxiety and migraine and there may be a shared underlying explanation as well. Anxiety is associated with negative outcomes such as migraine progression, medication overuse, stigma, and migraine-related disability [91–93, 100–105]. These results could have implications for the multidisciplinary management of anxiety in migraine both pharmacological and non-pharmacological and thereby increased relevance in complex treatment management across disciplines.

### Limitations and Strengths

A positive aspect of the review is the relatively large number of studies included in the review. There is a quite large amount of literature devoted to this issue. We have verified that focusing on the relationship of anxiety disorders and related problems in migraine is a fascinating area of literature. The purpose of this study was to review the literature on the relationship between migraine and concepts associated with anxiety and fear. Findings continue to support the importance of screening and diagnosis of anxiety disorders in migraine clinical care and referral and/or treatment as appropriate which may include psychotherapeutic approaches in the management of migraine.

What we can perceive as limits of this review is that we searched for inhomogeneous studies regarding design, and we could then only describe the results narratively. We did not focus on bias; the review does not synthesize and critically evaluate the results of the studies. This is not possible due to the non-uniform design. Because both qualitative and quantitative studies were included, the results cannot be easily generalized. Another limitation may be the selection of publications only in the English language, which may have omitted other important studies in non-English publications. Another limitation may be that we focused on only selected databases in our search strategy.

Further research needs to be done examining which approaches are the most effective for which patients in managing anxiety in migraine. The full range of potential empirically supported therapeutic approaches should be considered on a case by case basis.

### Clinical implications

These results suggest that screening and more comprehensive evaluation of anxiety comorbidity and associated symptoms and behaviors in patients with migraine using psychological diagnostic tools is important [94]. For patients with anxiety and fear, a comprehensive management approach of pharmacological [95, 96] and non-pharmacological treatment [75], including psychotherapy [91–93, 96] should be considered. Interventions for prevention of the development of anxiety disorders, symptoms and behaviors in patients with migraine is also important.

## Conclusions

Migraine, anxiety, and fear and avoidance behaviors have great comorbidity and coccurence cephalaphobia wenurrence and are associated with several undesirable outcomes. The relationships between anxiety disorders, anxious symptomatology and related behaviors are associated with decreased functioning and quality of life. Anxiety plays a significant role in exacerbating pain experiences by worsening the fear of pain and encouraging cognitive appraisal and catastrophic thinking. It heightens hypervigilance and pain intensity while driving behaviors aimed at avoiding potential harm, which reinforces the cycle as relief from anxiety strengthens avoidance. This cycle contributes to the overuse of acute medications, increases focus on pain triggers or avoidance of them, and lowers pain tolerance and pain threshold. Additionally, anxiety amplifies reactivity to pain anticipation, contributes to the development of specific phobias like cogniphobia (fear of thinking) and kinesiophobia (fear of movement), and intensifies an individual’s anxiety. Diagnostic vigilance for the presence of anxiety disorders as well as screening for anxious symptomology, and avoidance behaviors, and offering education, intervention and/or referrals as appropriate may be associated with improved clinical outcomes and quality of life. This is where CBT proves to be effective. The main premise of CBT is that the way we perceive ourselves, the world, the future, and think about events in our lives affects how we feel. And not only physically, but of course also emotionally. If it is possible to change the way we think about ourselves, the world, the future and things, then we can change the way we feel. [98, 99].

## Data Availability

No datasets were generated or analysed during the current study.

## Abbreviations

HPA axis: Hypothalamic-pituitary-adrenal
OCD: Obsessive-compulsive disorder
FA model: Fear-avoidance model
AE model: Avoidance-endurance model
TCI: Cloninger's temperament and character inventory
CM: Chronic migraine
EM: Episodic migraine
CS-HD: Cogniphobia Scale for Headache Disorders
HTSAQ: Headache Trigger Sensitivity and Avoidance Questionnaire
HAQ: The Headache Acceptance Questionnaire

## References

[1] Safiri S, Pourfathi H, Eagan A, Mansournia MA, Khodayari MT, Sullman MJM, Kaufman J, Collins G, Dai H, Bragazzi NL, Kolahi AA. Global, regional, and national burden of migraine in 204 countries and territories, 1990 to 2019. Pain. 2022;163(2):e293–309. 10.1097/j.pain.0000000000002275. 34001771 10.1097/j.pain.0000000000002275

[2] Sayers J. The world health report 2001-Mental health: new understanding, new hope. Bull World Health Organ. 2001;79(11):1085.

[3] Raggi A, Giovannetti AM, Quintas R, D’Amico D, Cieza A, Sabariego C, Bickenbach JE, Leonardi M. A systematic review of the psychosocial difficulties relevant to patients with migraine. J Headache Pain. 2012;13(8):595–606. 10.1007/s10194-012-0482-1. 23001069 10.1007/s10194-012-0482-1 PMC3484254

[4] Dresler T, Caratozzolo S, Guldolf K, Huhn JI, Loiacono C, Niiberg-Pikksööt T, Puma M, Sforza G, Tobia A, Ornello R, Serafini G. European Headache Federation School of Advanced Studies (EHF-SAS) Understanding the nature of psychiatric comorbidity in migraine: a systematic review focused on interactions and treatment implications. J Headache Pain. 2019;20(1):51. 10.1186/s10194-019-0988-x. 31072313 10.1186/s10194-019-0988-x PMC6734261

[5] Philips HC, Jahanshahi M. The components of pain behaviour report. Behav Res Ther. 1986;24(2):117–25. 10.1016/0005-7967(86)90082-3. 3964177 10.1016/0005-7967(86)90082-3

[6] Peres MF, Mercante JP, Tobo PR, Kamei H, Bigal ME. Anxiety and depression symptoms and migraine: a symptom-based approach research. J Headache Pain. 2017;18(1):37. 10.1186/s10194-017-0742-1. 28324317 10.1186/s10194-017-0742-1 PMC5360747

[7] Smitherman TA, Penzien DB, Maizels M. Anxiety disorders and migraine intractability and progression. Curr Pain Headache Rep. 2008;12(3):224–9. 10.1007/s11916-008-0039-9. 18796274 10.1007/s11916-008-0039-9

[8] Buse DC, Reed ML, Fanning KM, Bostic R, Dodick DW, Schwedt TJ, Munjal S, Singh P, Lipton RB. Comorbid and co-occurring conditions in migraine and associated risk of increasing headache pain intensity and headache frequency: results of the migraine in America symptoms and treatment (MAST) study. J Headache Pain. 2020;21(1):23. 32122324 10.1186/s10194-020-1084-y PMC7053108

[9] Rogers DG, Protti TA, Smitherman TA. Fear, avoidance, and disability in headache disorders. Curr Pain Headache Rep. 2020;29:33. 10.1007/s11916-020-00865-9. 10.1007/s11916-020-00865-9 32472171

[10] Smitherman TA, Kolivas ED, Bailey JR. Panic disorder and migraine: comorbidity, mechanisms, and clinical implications. Headache. 2013;53(1):23–45. 10.1111/head.12004. 23278473 10.1111/head.12004

[11] Minen MT, Begasse De Dhaem O, Kroon Van Diest A, Powers S, Schwedt TJ, Lipton R, Silbersweig D. Migraine and its psychiatric comorbidities. J Neurol Neurosurg Psychiatry. 2016;87(7):741–9. 26733600 10.1136/jnnp-2015-312233

[12] Luo J. Association between migraine and anxiety symptoms: Results from the study of women’s health across the nation. J Affect Disord. 2021;295:1229–33. 34706437 10.1016/j.jad.2021.09.036

[13] Blau JN. Fears aroused in patients by migraine. Br Med J (Clin Res Ed). 1984;288(6424):1126. 10.1136/bmj.288.6424.1126. 6424760 10.1136/bmj.288.6424.1126 PMC1441408

[14] Rutberg S, Öhrling K. Migraine – more than a headache: women’s experiences of living with migraine. Disabil Rehabil. 2012;34(4):329–36. 10.3109/09638288.2011.607211. 21981545 10.3109/09638288.2011.607211 PMC3267523

[15] Scher AI, Midgette LA, Lipton RB. Risk factors for headache chronification. Headache. 2007;48(1):16–25. 10.1111/j.1526-4610.2007.00970.x. 10.1111/j.1526-4610.2007.00970.x 18184281

[16] Lantéri-Minet M, Radat F, Chautard MH, Lucas C. Anxiety and depression associated with migraine: Influence on migraine subjectsʼ disability and quality of life, and acute migraine management. Pain. 2005;118(3):319–26. 10.1016/j.pain.2005.09.010. 16289799 10.1016/j.pain.2005.09.010

[17] Pesa J, Lage MJ. The medical costs of migraine and comorbid anxiety and depression. Headache. 2004;44(6):562–70. 10.1111/j.1526-4610.2004.446004.x. 15186300 10.1111/j.1526-4610.2004.446004.x

[18] Guidetti V, Galli F, Fabrizi P, Giannantoni AS, Napoli L, Bruni O, Trillo S. Headache and psychiatric comorbidity: clinical aspects and outcome in an 8-year follow-up study. Cephalalgia. 1998;18(7):455–62. 10.1046/j.1468-2982.1998.1807455.x. 9793697 10.1046/j.1468-2982.1998.1807455.x

[19] Buse DC, Greisman JD, Baigi K, Lipton RB. Migraine progression: a systematic review. Headache. 2019;59(3):306–38. 10.1111/head.13459. 30589090 10.1111/head.13459

[20] Reme SE. Anxiety could play a larger role than depression in migraine headache. Scand J Pain. 2016;13(1):127. 10.1016/j.sjpain.2016.08.002. 28850510 10.1016/j.sjpain.2016.08.009

[21] Radat F, Swendsen J. Psychiatric comorbidity in migraine: a review. Cephalalgia. 2005;25(3):165–78. 10.1111/j.1468-2982.2004.00839.x. 15689191 10.1111/j.1468-2982.2004.00839.x

[22] Breslau N. Psychiatric comorbidity in migraine. Cephalalgia. 1998;18(Suppl 22):56–8. 9793713 10.1177/0333102498018s2210

[23] Buse DC, Manack A, Serrano D, Turkel C, Lipton RB. Sociodemographic and comorbidity profiles of chronic migraine and episodic migraine sufferers. J Neurol Neurosurg Psychiatry. 2010;81(4):428–32. 20164501 10.1136/jnnp.2009.192492

[24] Buse DC, Reed ML, Fanning KM, Bostic RC, Lipton RB. Demographics, headache features, and comorbidity profiles in relation to headache frequency in people with migraine: results of the american migraine prevalence and prevention (AMPP) study. Headache. 2020. 10.1111/head.13966. 33090481 10.1111/head.13966

[25] Buse DC, Reed ML, Fanning KM, Bostic R, Dodick DW, Schwedt TJ, Munjal S, Singh P, Lipton RB. Comorbid and co-occurring conditions in migraine and associated risk of increasing headache pain intensity and headache frequency: results of the migraine in America symptoms and treatment (MAST) study. J Headache Pain. 2020;21(1):23. 10.1186/s10194-020-1084-y. 32122324 10.1186/s10194-020-1084-y PMC7053108

[26] Breslau N, Schultz LR, Stewart WF, Lipton R, Welch KM. Headache types and panic disorder: directionality and specificity. Neurology. 2001;56(3):350–4. 10.1212/wnl.56.3.350. 11171900 10.1212/wnl.56.3.350

[27] Breslau N, Davis GC, Andreski P. Migraine, psychiatric disorders, and suicide attempts: An epidemiologic study of young adults. Psychiatry Res. 1991;37(1):11–23. 10.1016/0165-1781(91)90102-u. 1862159 10.1016/0165-1781(91)90102-u

[28] Breslau N, Davis GC. Migraine, physical health and psychiatric disorder: A prospective epidemiologic study in young adults. J Psychiatr Res. 1993;27(2):211–21. 10.1016/0022-3956(93)90009-q. 8366470 10.1016/0022-3956(93)90009-q

[29] Zhang M, Liu Y, Zhao M, Tang W, Wang X, Dong Z, Yu S. Depression and anxiety behaviour in a rat model of chronic migraine. J Headache Pain. 2017;18(1):27. 10.1186/s10194-017-0736-z. 28224378 10.1186/s10194-017-0736-z PMC5319946

[30] Green MW. Headaches: psychiatric aspects. Neurol Clin. 2011;29(1):65–vii. 10.1016/j.ncl.2010.10.004. 21172571 10.1016/j.ncl.2010.10.004

[31] Baldacci F, Lucchesi C, Cafalli M, Poletti M, Ulivi M, Vedovello M, Giuntini M, Mazzucchi S, Del Prete E, Vergallo A, Nuti A, Gori S. Migraine features in migraineurs with and without anxiety-depression symptoms: a hospital-based study. Clin Neurol Neurosurg. 2015;132:74–8. 10.1016/j.clineuro.2015.02.017. 25804622 10.1016/j.clineuro.2015.02.017

[32] Noseda R, Kainz V, Borsook D, Burstein R. Neurochemical pathways that converge on thalamic trigeminovascular neurons: potential substrate for modulation of migraine by sleep, food intake, stress and anxiety. PLoS ONE. 2014;9(8):e103929. 10.1371/journal.pone.0103929. 25090640 10.1371/journal.pone.0103929 PMC4121288

[33] Vlaeyen JWS, Crombez G, Linton SJ. The fear-avoidance model of pain. Pain. 2016;157(8):1588–9. 10.1097/j.pain.0000000000000574. 27428892 10.1097/j.pain.0000000000000574

[34] Mittinty MM, McNeil DW, Brennan DS, Randall CL, Mittinty MN, Jamieson L. Assessment of pain-related fear in individuals with chronic painful conditions. J Pain Res. 2018;11:3071–7. 10.2147/JPR.S163751. 30555253 10.2147/JPR.S163751 PMC6280906

[35] Chen Z, Chen X, Liu M, Ma L, Yu S. Lower hippocampal subfields volume in relation to anxiety in medication-overuse headache. Mol Pain. 2018;14:174480691876125. 10.1177/1744806918761257. 10.1177/1744806918761257 PMC5871043 29424272

[36] Helsen K, Leeuw M, Vlaeyen J. Fear and Pain. In: Gebhart GF, Schmidt RF, editors. Encyclopedia of Pain. Berlin: Springer; 2013. p. 1261–7.

[37] Zale EL, Lange KL, Fields SA, Ditre JW. The relation between pain-related fear and disability: a meta-analysis. J Pain. 2013;14(10):1019–30. 10.1016/j.jpain.2013.05.005. 23850095 10.1016/j.jpain.2013.05.005 PMC3791167

[38] Asmundson GJ, Norton PJ, Veloso F. Anxiety sensitivity and fear of pain in patients with recurring headaches. Behav Res Ther. 1999;37(8):703–13. 10.1016/s0005-7967(98)00172-7. 10452173 10.1016/s0005-7967(98)00172-7

[39] Hursey KG, Jacks SD. Fear of pain in recurrent headache sufferers. Headache. 1992;32(6):283–6. 10.1111/j.1526-4610.1992.hed3206283.x. 1399548 10.1111/j.1526-4610.1992.hed3206283.x

[40] Ruscheweyh R, Pereira D, Hasenbring MI, Straube A. Pain-related avoidance and endurance behaviour in migraine: an observational study. J Headache Pain. 2019;20(1):9. 10.1186/s10194-019-0962-7. 30658566 10.1186/s10194-019-0962-7 PMC6734268

[41] Rogers DG, Protti TA, Smitherman TA. Fear, avoidance, and disability in headache disorders. Curr Pain Headache Rep. 2020;24:33. 10.1007/s11916-020-00865-9. 32472171 10.1007/s11916-020-00865-9

[42] van Tulder MW, Ostelo R, Vlaeyen JW, Linton SJ, Morley SJ, Assendelft WJ. Behavioral treatment for chronic low back pain: a systematic review within the framework of the Cochrane Back Review Group. Spine. 2000;25(20):2688–99. 10.1097/00007632-200010150-00024. 11034658 10.1097/00007632-200010150-00024

[43] Hasenbring M, Verbunt JA. Fear-avoidance and endurance-related responses to pain: new models of behavior and their consequences for clinical practice. Clin J Pain. 2010;26(9):747–53. 10.1097/AJP.0b013e3181e104f2. 20664333 10.1097/AJP.0b013e3181e104f2

[44] Black AK, Fulwiler JC, Smitherman TA. The role of fear of pain in headache. Headache. 2015;55(5):669–79. 10.1111/head.12561. 25903510 10.1111/head.12561

[45] Mongini F, Fassino S, Rota E, Deregibus A, Levi M, Monticone D, Abbate-Daga G. The temperament and character inventory in women with migraine. J Headache Pain. 2005;6(4):247–9. 10.1007/s10194-005-0198-6. 16362677 10.1007/s10194-005-0198-6 PMC3452018

[46] Anxiety sensitivity. In: Harvard Health Publishing. 2014. https://www.health.harvard.edu/newsletter_article/Anxiety_sensitivity. Accessed:15 Jan 2023.

[47] Farris SG, Burr EK, Abrantes AM, Thomas JG, Godley FA, Roth JL, Lipton RB, Pavlovic JM, Bond DS. Anxiety sensitivity as a risk indicator for anxiety, depression, and headache severity in women with migraine. Headache. 2019;59(8):1212–20. 10.1111/head.13568. 31166015 10.1111/head.13568

[48] Farris SG, Thomas JG, Abrantes AM, Lipton RB, Burr EK, Godley FA, Roth JL, Pavlovic JM, Bond DS. Anxiety sensitivity and intentional avoidance of physical activity in women with probable migraine. Cephalalgia. 2019;39(11):1465–9. 10.1177/0333102419861712. 31260336 10.1177/0333102419861712

[49] Asmundson GJ, Taylor S. Role of anxiety sensitivity in pain-related fear and avoidance. J Behav Med. 1996;19(6):577–86. 10.1007/bf01904905. 8970916 10.1007/BF01904905

[50] Norton PJ, Asmundson GJ. Anxiety sensitivity, fear, and avoidance behavior in headache pain. Pain. 2004;111(1–2):218–23. 10.1016/j.pain.2004.06.018. 15327826 10.1016/j.pain.2004.06.018

[51] Smitherman TA, Davis RE, Walters AB, Young J, Houle TT. Anxiety sensitivity and headache: diagnostic differences, impact, and relations with perceived headache triggers. Cephalalgia. 2014;35(8):710–21. 10.1177/0333102414557840. 25352500 10.1177/0333102414557840

[52] Fiske DW. Consistency of the factorial structures of personality ratings from different add to the citing literature sources. Psychol Sci Public Interest. 1949;44(3):329–44. 10.1037/h0057198. 10.1037/h0057198 18146776

[53] Davis RE, Smitherman TA, Baskin SM. Personality traits, personality disorders, and migraine: a review. Neurol Sci. 2013;34(1):7–10. 10.1007/s10072-013-1379-8. 10.1007/s10072-013-1379-8 23695036

[54] Balottin U, Fusar Poli P, Termine C, Molteni S, Galli F. Psychopathological symptoms in child and adolescent migraine and tension-type headache: a meta-analysis. Cephalalgia. 2012;33(2):112–22. 10.1177/0333102412468386. 23203505 10.1177/0333102412468386

[55] Lake AE, Rains JC, Penzien DB, Lipchik GL. Headache and psychiatric comorbidity: historical context, clinical implications, and research relevance. Headache. 2005;45(5):493–506. 10.1111/j.1526-4610.2005.05101.x. 15953266 10.1111/j.1526-4610.2005.05101.x

[56] Rothrock J, Lopez I, Zweilfer R, Andress-Rothrock D, Drinkard R, Walters N. Borderline personality disorder and migraine. Headache. 2007;47(1):22–6. 10.1111/j.1526-4610.2007.00649.x. 17355490 10.1111/j.1526-4610.2007.00649.x

[57] Yang F, Dos Santos IAM, Gomez RS, Kummer A, Barbosa IG, Teixeira AL. Personality disorders are associated with more severe forms of migraine. Acta Neurol Belg. 2019;119(2):201–5. 10.1007/s13760-018-1050-5. 30474829 10.1007/s13760-018-1050-5

[58] Saper JR, Lake AE 3rd. Borderline personality disorder and the chronic headache patient: review and management recommendations. Headache. 2002;42(7):663–74. 10.1046/j.1526-4610.2002.02156.x 10.1046/j.1526-4610.2002.02156.x 12482221

[59] Schwedt TJ, Buse DC, Argoff CE, Reed ML, Fanning KM, Hussar CR, Adams AM, Lipton RB. Medication overuse and headache burden: results from the cameo study. Neurol Clin Pract. 2021;11(3):216–26. 10.1212/CPJ.0000000000001037. 34476122 10.1212/CPJ.0000000000001037 PMC8382341

[60] Sances G, Galli F, Anastasi S, Ghiotto N, De Giorgio G, Guidetti V, Firenze C, Pazzi S, Quartesan R, Gallucci M, Nappi G. Medication-overuse headache and personality: a controlled study by means of the MMPI-2. Headache. 2010;50(2):198–209. 10.1111/j.1526-4610.2009.01593.x. 20039955 10.1111/j.1526-4610.2009.01593.x

[61] Bottiroli S, Galli F, Viana M, Sances G, Tassorelli C. Traumatic experiences, stressful events, and alexithymia in chronic migraine with medication overuse. Front Psychol. 2018;9:704. 10.3389/fpsyg.2018.00704. 29867669 10.3389/fpsyg.2018.00704 PMC5960722

[62] Galli F, Caputi M, Sances G, Vegni E, Bottiroli S, Nappi G, Tassorelli C. Alexithymia in chronic and episodic migraine: a comparative study. J Ment Health. 2017;26(3):192–6. 10.3109/09638237.2015.1124404. 26732465 10.3109/09638237.2015.1124404

[63] Frith U. Emanuel Miller lecture: Confusions and controversies about Asperger syndrome. J Child Psychol Psychiatry. 2004;45(4):672–86. 10.1111/j.1469-7610.2004.00262.x. 15056300 10.1111/j.1469-7610.2004.00262.x

[64] Tani P, Lindberg N, Joukamaa M, Nieminen-von Wendt T, von Wendt L, Appelberg B, Rimón R, Porkka-Heiskanen T. Asperger syndrome, alexithymia and perception of sleep. Neuropsychobiology. 2004;49(2):64–70. 10.1159/000076412. 14981336 10.1159/000076412

[65] Merikangas KR, Angst J, Isler H. Migraine and psychopathology: results of the Zurich cohort study of young adults. Arch Gen Psychiatry. 1990;47(9):849–53. 10.1001/archpsyc.1990.01810210057008. 2393343 10.1001/archpsyc.1990.01810210057008

[66] Peroutka SJ, Price SC, Wilhoit TL, Jones KW. Comorbid migraine with aura, anxiety, and depression isassociated with dopamine D2 receptor (DRD2) NcoI alleles. Mol Med. 1998;4(1):14–21. 10.1007/BF03401725. 9513185 PMC2230268

[67] Swartz KL, Pratt LA, Armenian HK, Lee LC, Eaton WW. Mental disorders and the incidence of migraine headaches in a community sample: results from the Baltimore epidemiologic catchment area follow-up study. Arch Gen Psychiatry. 2000;57(10):945. 10.1001/archpsyc.57.10.945. 11015812 10.1001/archpsyc.57.10.945

[68] Peres MF, Mercante JP, Guendler VZ, Corchs F, Bernik MA, Zukerman E, Silberstein SD. Cephalalgiaphobia: a possible specific phobia of illness. J Headache Pain. 2007;8(1):56–9. 10.1007/s10194. 17361383 10.1007/s10194-007-0361-3 PMC3476114

[69] Giannini G, Zanigni S, Grimaldi D, Melotti R, Pierangeli G, Cortelli P, Cevoli S. Cephalalgiaphobia as a feature of high-frequency migraine: a pilot study. J Headache Pain. 2013;14(1):1–6. 10.1186/1129-2377-14-49. 23759110 10.1186/1129-2377-14-49 PMC3686604

[70] Miller TW, Kraus RF. An overview of chronic pain. Hosp Community Psychiatry. 1990;41(4):433–40. 2185147 10.1176/ps.41.4.433

[71] Martelli MF, Grayson RL, Zasler ND. Posttraumatic headache: neuropsychological and psychological effects and treatment implications. J Head Trauma Rehabil. 1999;14(1):49–69. 9949246 10.1097/00001199-199902000-00007

[72] Seng EK. Cogniphobia in Migraine: Fear and Avoidance of Thinking. In: Neurology advisor. 2017. https://www.neurologyadvisor.com/topics/migraine-and-headache/cogniphobia-in-migraine-the-fear-and-avoidance-of-thinking/. Accessed 15 Jan 2023.

[73] Todd DD, Martelli MF, Grayson RL. The cogniphobia scale (C-Scale): a measure of headache impact. Test in the public domain. 1998. http://villamartelli.com/nanposters1999.htm.

[74] Seng EK, Klepper JE. Development of the Cogniphobia Scale for headache disorders (CS-HD): a pilot study. Psychol Assess. 2017;29(10):1296–301. 10.1037/pas0000432. 28125248 10.1037/pas0000432 PMC5529280

[75] Martin PR, Reece J, Callan M, MacLeod C, Kaur A, Gregg K, Goadsby PJ. Behavioral management of the triggers of recurrent headache: a randomized controlled trial. Behav Res Ther. 2014;61:1–11. 10.1016/j.brat.2014.07.002. 25108482 10.1016/j.brat.2014.07.002

[76] Martin PR. Behavioral management of migraine headache triggers: learning to cope with triggers. Curr Pain Headache Rep. 2010;14(3):221–7. 10.1007/s11916-010-0112-z. 20425190 10.1007/s11916-010-0112-z

[77] Suhr J, Spickard B. Pain-related fear is associated with cognitive task avoidance: exploration of the cogniphobia construct in a recurrent headache sample. Clin Neuropsychol. 2012;26(7):1128–41. 10.1080/13854046.2012.713121. 22928643 10.1080/13854046.2012.713121

[78] Karimi L, Wijeratne T, Crewther SG, Evans AE, Ebaid D, Khalil H. The migraine-anxiety comorbidity among migraineurs: a systematic review. Front Neurol. 2021;11:613372. 10.3389/fneur.2020.613372. 33536997 10.3389/fneur.2020.613372 PMC7848023

[79] Kumar R, Asif S, Bali A, Dang AK, Gonzalez DA. The development and impact of anxiety with migraines: a narrative review. Cureus. 2022;14(6):e26419. 10.7759/cureus.26419. 35923673 10.7759/cureus.26419 PMC9339341

[80] Klan T, Bräscher AK, Klein S, Diezemann-Prößdorf A, Guth AL, Gaul C, Witthöft M. Assessing attack-related fear in headache disorders-Structure and psychometric properties of the Fear of Attacks in Migraine Inventory. Headache. 2022;62(3):294–305. 10.1111/head.14272. 35181884 10.1111/head.14272

[81] Kubik SU, Martin PR. The Headache Triggers Sensitivity and Avoidance Questionnaire: Establishing the Psychometric Properties of the Questionnaire. Headache. 2017;57(2):236–54. 27753075 10.1111/head.12940

[82] Caroli A, Klan T, Gaul C, Kubik SU, Martin PR, Witthöft M. Types of triggers in migraine - factor structure of the headache triggers sensitivity and avoidance questionnaire and development of a new short form (HTSAQ-SF). Headache. 2020;60(9):1920–9. 10.1111/head.13896. 32654136 10.1111/head.13896

[83] Hamer JD, Sackey ET, Maack DJ, Smitherman TA. Development of a measure to assess acceptance of headache: The Headache Acceptance Questionnaire (HAQ). Cephalalgia. 2020;40(8):797–807. 10.1177/0333102420907596. 32070128 10.1177/0333102420907596

[84] Spitzer RL, Kroenke K, Williams JB, Löwe B. A brief measure for assessing generalized anxiety disorder: the GAD-7. Arch Intern Med. 2006;166:1092–7. 10.1001/archinte.166.10.1092. 16717171 10.1001/archinte.166.10.1092

[85] Kroenke K, Spitzer RL, Williams JB, Löwe B. An ultra-brief screening scale for anxiety and depression: the PHQ-4. Psychosomatics. 2009;50(6):613–21. 10.1176/appi.psy.50.6.613. 19996233 10.1176/appi.psy.50.6.613

[86] Lipton RB, Fanning KM, Buse DC, Martin VT, Hohaia LB, Adams AM, Reed ML, Goadsby PJ. Migraine progression in subgroups of migraine based on comorbidities: results of the CaMEO study. Neurology. 2019;93:e2224–36. 31690685 10.1212/WNL.0000000000008589 PMC6937494

[87] Hamilton M. The assessment of anxiety states by rating. Br J Med Psychol. 1959;32:50–5. 13638508 10.1111/j.2044-8341.1959.tb00467.x

[88] Zigmond AS, Snaith RP. The hospital anxiety and depression scale. Acta Psychiatr Scand. 1983;67:361–70. 10.1111/j.1600-0447.1983.tb09716.x. 6880820 10.1111/j.1600-0447.1983.tb09716.x

[89] Alvarez-Astorga A, García-Azorín D, Hernández M, de la Red H, Sotelo E, Uribe F, Guerrero AL. Pain catastrophising in a population of patients with migraine. Neurologia (Engl Ed). 2021;36(1):24–28. English, Spanish. 10.1016/j.nrl.2018.10.005 10.1016/j.nrl.2018.10.005 30857787

[90] Sullivan MJL, Bishop SR, Pivik J. The pain catastrophizing scale: development and validation. Psychol Assess. 1995;7(4):524–32. 10.1037/1040-3590.7.4.524.

[91] Seng EK, Shapiro RE, Buse DC, Robbins MS, Lipton RB, Parker A. The unique role of stigma in migraine-related disability and quality of life. Headache. 2022;62(10):1354–2136. 36321956 10.1111/head.14401

[92] Shapiro RE, Nicholson RA, Seng EK, Buse DC, Reed ML, Zagar AJ, Ashina S, Muenzel EJ, Hutchinson S, Pearlman EM, Lipton RB. Migraine-related stigma and its relationship to disability, interictal burden, and quality of life: results of the OVERCOME (US) Study. Neurology. 2024;102(3):e208074. 38232340 10.1212/WNL.0000000000208074 PMC11097761

[93] Lipton RB, Seng EK, Chu MK, Reed ML, Fanning KM, Adams AM, Buse DC. The effect of psychiatric comorbidities on headache-related disability in migraine: results from the chronic migraine epidemiology and outcomes (CaMEO) Study. Headache. 2020;60(8):1683–96. 33448374 10.1111/head.13914 PMC7496280

[94] Seng EK, Buse DC, Klepper JE, Mayson JS, Grinberg AS, Grosberg BM, Pavlovic JM, Robbins MS, Vollbracht SE, Lipton RB. Psychological factors associated with chronic migraine and severe migraine-related disability: an observational study in a tertiary headache center. Headache. 2017;57(4):593–604. 10.1111/head.13021. 28139000 10.1111/head.13021 PMC5378650

[95] Lipton RB, Buse DC, Dodick DW, Schwedt TJ, Singh P, Munjal S, Fanning K, Bostic Bs R, Reed ML. Burden of increasing opioid use in the treatment of migraine: Results from the Migraine in America Symptoms and Treatment Study. Headache. 2021;61(1):103–16. 33326608 10.1111/head.14018

[96] Schwedt TJ, Alam A, Reed ML, Fanning KM, Munjal S, Buse DC, Dodick DW, Lipton RB. Factors associated with acute medication overuse in people with migraine: results from the 2017 migraine in America symptoms and treatment (MAST) study. J Headache Pain. 2018;19(1):38. 29797100 10.1186/s10194-018-0865-z PMC5968010

[97] Yalug I, Selekler M, Erdogan A, Kutlu A, Dundar G, Ankarali H, Aker T. Correlations between alexithymia and pain severity, depression, and anxiety among patients with chronic and episodic migraine. Psychiatry Clin Neurosci. 2010;64(3):231–8. 10.1111/j.1440-1819.2010.02093.x. 20602723 10.1111/j.1440-1819.2010.02093.x

[98] Beehler GP, Conrad ML, Dimoff J, Haslam A Murphy J. Brief cognitive behavioral therapy for chronic pain: patient guidebook. Washington, DC: U.S. Department of Veterans Affairs; 2021.

[99] Thorn, BE. Cognitive therapy for chronic pain, a step-by-step guide. New York, NY: Guilford Press; 2017.

[100] Rogers DG, Protti TA, Smitherman TA. Fear, Avoidance, and Disability in Headache Disorders. Curr Pain Headache Rep. 2020;24(7):33. 10.1007/s11916-020-00865-9. 32472171 10.1007/s11916-020-00865-9

[101] Black AK, Fulwiler JC, Smitherman TA. The role of fear of pain in headache. Headache. 2015;55(5):669–79. 10.1111/head.12561. 25903510 10.1111/head.12561

[102] Smitherman TA, Davis RE, Walters AB, Young J, Houle TT. Anxiety sensitivity and headache: diagnostic differences, impact, and relations with perceived headache triggers. Cephalalgia. 2015;35(8):710–21. 10.1177/0333102414557840. 25352500 10.1177/0333102414557840

[103] Buse DC, Reed ML, Fanning KM, Bostic R, Dodick DW, Schwedt TJ, Munjal S, Singh P, Lipton RB. Comorbid and co-occurring conditions in migraine and associated risk of increasing headache pain intensity and headache frequency: results of the migraine in America symptoms and treatment (MAST) study. J Headache Pain. 2020;21(1):23. 10.1186/s10194-020-1084-y. 32122324 10.1186/s10194-020-1084-y PMC7053108

[104] Buse DC, Silberstein SD, Manack AN, Papapetropoulos S, Lipton RB. Psychiatric comorbidities of episodic and chronic migraine. J Neurol. 2013;260(8):1960–9. 10.1007/s00415-012-6725-x. 23132299 10.1007/s00415-012-6725-x

[105] Minen MT, Begasse De Dhaem O, Kroon Van Diest A, Powers S, Schwedt TJ, Lipton R, Silbersweig D. Migraine and its psychiatric comorbidities. J Neurol Neurosurg Psychiatry. 2016;87(7):741–9. 10.1136/jnnp-2015-312233. 26733600 10.1136/jnnp-2015-312233

